# Enhanced inhibition of influenza virus infection by peptide–noble-metal nanoparticle conjugates

**DOI:** 10.3762/bjnano.10.104

**Published:** 2019-05-14

**Authors:** Zaid K Alghrair, David G Fernig, Bahram Ebrahimi

**Affiliations:** 1 Department of Biochemistry Institute of Integrative Biology, Biosciences Building, Crown Street, University of Liverpool, Liverpool, L69 7ZB, UK,; 2 Department of Functional and Comparative Genomics, Institute of Integrative Biology, Biosciences Building, Crown Street, University of Liverpool, Liverpool, L69 7ZB, UK

**Keywords:** antiviral peptides, gold nanoparticles, influenza virus, lytic infection, silver nanoparticles

## Abstract

The influenza (“flu”) type-A virus is a major medical and veterinary health concern and causes global pandemics. The peptide “FluPep” is an established inhibitor of influenza virus infectivity in model systems. We have explored the potential for noble-metal nanoparticle conjugates of FluPep to enhance its antiviral activity and to determine their potential as a delivery platform for FluPep. FluPep ligand is FluPep extended at its N-terminus with the sequence CVVVTAAA, to allow for its incorporation into a mixed-matrix ligand shell of a peptidol and an alkanethiol ethylene glycol consisting of 70% CVVVTol and 30% HS(CH_2_)_11_(OC_2_H_4_)_4_OH (mol/mol). Gold and silver nanoparticles (ca. 10 nm diameter) with up to 5% (mol/mol) FluPep ligand remained as stable as the control of mixed-matrix-passivated nanoparticles in a variety of tests, including ligand exchange with dithiothreitol. The free FluPep ligand peptide was found to inhibit viral plaque formation in canine MDCK cells (IC_50_ = 2.1 nM), but was less potent than FluPep itself (IC_50_ = 140 pM). Nanoparticles functionalised with FluPep ligand showed enhanced antiviral activity compared to the free peptides. The IC_50_ value of the FluPep-functionalised nanoparticles decreased as the grafting density of FluPep ligand increased from 0.03% to 5% (both mol/mol), with IC_50_ values down to about 10% of that of the corresponding free peptide. The data demonstrate that conjugation of FluPep to gold and silver nanoparticles enhances its antiviral potency; the antimicrobial activity of silver ions may enable the design of even more potent antimicrobial inhibitors, capable of targeting both influenza and bacterial co-infections.

## Introduction

The influenza (“flu”) type-A virus is a major health concern for humans and livestock animals. The primary mode of transmission is by the respiratory route. Flu infection occurs seasonally and can cause global pandemics, e.g., the 2009 H1N1 subtype swine influenza, which resulted in more than 18000 deaths worldwide [1]. The treatment of influenza infections is difficult, because the virus has a segmented RNA genome that has a high potential to recombine and create new strains through a mechanism termed re-assortment [2]. Other potential risks to human populations are the zoonotic avian (bird) and porcine (swine) influenza viruses. Vaccination remains the most effective means to prevent and control infection [3]. However, the lead time to vaccine production is around nine months, efficacy is not always complete, only a fraction of the human population is vaccinated, and although some vaccines have been trialled against avian influenza, farm animals in general are not routinely vaccinated on a global scale [4]. There is, therefore, the need for drugs to combat influenza infection in a more effective and timely manner.

Currently there are two common types of anti-influenza drugs, based on their mechanism of action. The first class are neuraminidase inhibitors such as Oseltamivir (Tamiflu). The second class are virus ion-channel blockers, such as Amantadine (Symetrel). The effectiveness of Tamiflu has been questioned [5], and in any case the emerging resistance of the influenza virus is leading to reduced effectiveness [6]. The promise of peptide-based antiviral drugs has been established by the approval of the Food and Drug Administration (USA) of Enfuvirtidie against HIV [7]. The potential therapeutic use of other antiviral peptides has been demonstrated in HIV [8–9], hepatitis C [10], herpes simplex [11–12], influenza virus [13–15].

The infectivity of influenza A viruses, including the H1N1 subtype, is strongly inhibited by a peptide called FluPep [15]. FluPep was originally identified as a sequence in tyrosine kinase inhibitor peptide (Tkip), thought to act as a mimic of the suppressor of cytokine signalling (SOCS) protein [16]. However, the antiviral activity of FluPep does not depend on blocking cytokine signalling, which is intracellular, but instead this peptide appears to exert its antiviral activity from the outside of the cell. Thus, the addition of FluPep to cells in culture prevents infection by influenza viruses, as does intranasal delivery of the peptide in a murine model of human influenza [15].

Noble-metal nanoparticles possess a strong plasmon absorbance, which allows for the detection at very low levels, using a range of approaches, from absorbance [17–18] to photothermal microscopy [19] and various extensions of the latter [20–21]. Noble-metal nanoparticles can be passivated and functionalised with biomolecules such that they possess the biological selectivity and specificity of the grafted biological functional entity [22], which includes peptides [23–24]. Presentation of a functional peptide by means of a nanoparticle has a number of advantages. Thus, nanoparticle conjugation may enhance the solubility of the peptide, as well as enhance the biological activity of the peptide, for example, due to multivalent functionalisation of the nanoparticles. In addition, silver possesses innate antimicrobial activities [25]. Thus, noble-metal nanoparticles are potentially useful as both functional probes for antiviral peptides and as therapeutic delivery platforms. We have, therefore, synthesized gold and silver nanoparticles functionalised with FluPep and analysed the anti-influenza activity of the nanoparticle–FluPep ligand conjugates. The results demonstrate that the nanoparticle–FluPep ligand conjugates reduce the infectivity of influenza virus with greater antiviral activity than the free peptide, making this a viable tool for the development of a peptide formulation that efficiently combats seasonal, pandemic, and zoonotic influenza infections.

## Results and Discussion

### Stability of FluPep functionalised gold nanoparticles

**Figure 1:**
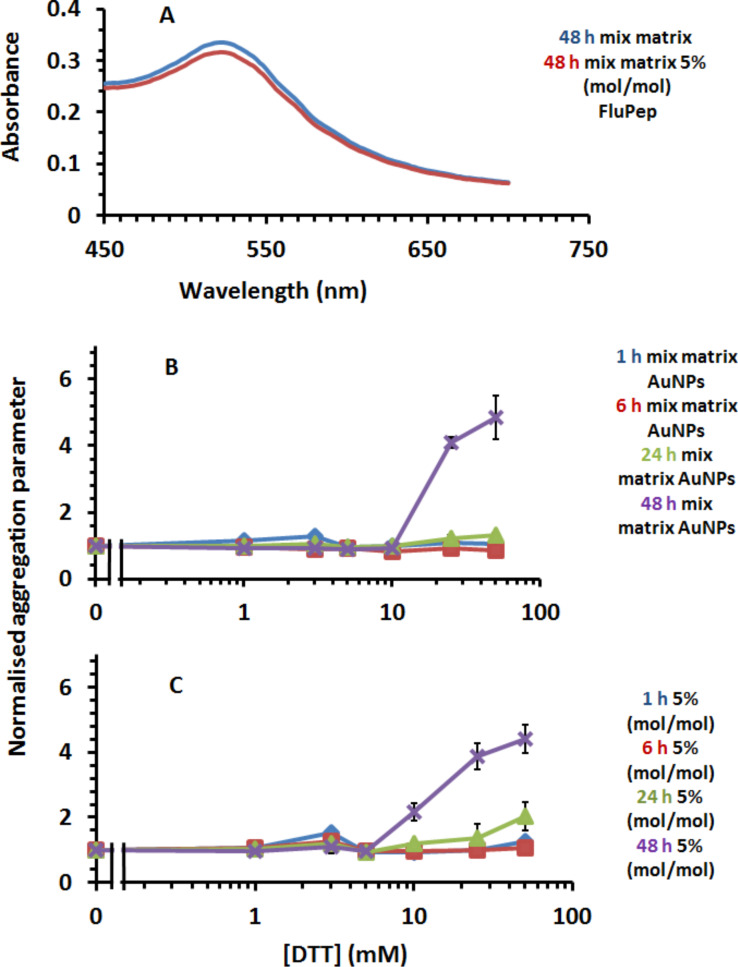
Stability of gold nanoparticles to DTT ligand exchange. (A) UV–vis spectra of mixed-matrix-capped gold nanoparticles and mixed-matrix-capped gold nanoparticles incorporating 5% (mol/mol) FluPep ligand in PBS. Time- and dose-dependence of DTT ligand exchange for (B) mixed-matrix gold nanoparticles and (C) gold nanoparticles with a ligand shell incorporating 5% (mol/mol) FluPep ligand. Results in (B) and (C) are the mean ± SD (*n* = 3).

The mixed-matrix ligand shell of 70:30 (mol/mol) peptidol and alkanethiol ethylene glycol assembled on gold nanoparticles has a well-characterised stability with respect to ligand exchange and non-specific binding [26–27], but the effect of incorporating the FluPep amino acid sequence at the C-terminus of the CVVVT matrix sequence was unknown. Since the molcular weight of FluPep ligand (2967 Da) is greater than that required for group separation on Sephadex G25 (1000 Da), additional purification by means of washes on a 10 kDa cut-off Nanosep filter were included to ensure removal of any free FluPep ligand. When up to 5% (mol/mol) FluPep ligand was incorporated in the ligand matrix, the gold nanoparticles still eluted in the void volume of the Sephadex G25 column, so did not bind non-specifically to this chromatography matrix, and their UV–vis spectrum in PBS was indistinguishable from that of control mixed-matrix gold nanoparticles (Figure 1A). This indicates that the FluPep sequence did not reduce the stability of the gold nanoparticles under these standard conditions. A more stressful test is ligand exchange with small thiols [26–29]. The ligand exchange results in a ligand shell that is unable to prevent electrolyte-induced aggregation of the nanoparticles, demonstrated by a decrease in the plasmon absorption at 520 nm.

Gold nanoparticles with a ligand shell incorporating 5% (mol/mol) FluPep ligand had a very similar resistance to ligand exchange with DTT as the control mixed-matrix-protected gold nanoparticles. Their aggregation parameter was unchanged up to 5 mM DTT, even after 48 h incubation (Figure 1B,C). At 10 mM DTT after 48 h there was some evidence for ligand exchange, as the aggregation parameter was above 1.0 and at 25 mM DTT the ligand shell was clearly compromised. Nanoparticles incorporating lesser amounts of FluPep ligand (0.1% to 3% (mol/mol)) were no less stable (Supporting Information File 1, Figure S1A–F). Consequently, the incorporation of up to 5% (mol/mol) FluPep ligand in the ligand mixture did not reduce the stability of the gold nanoparticles with respect to ligand exchange and such nanoparticles could be used in cell culture medium.

### Purification of functionalised gold nanoparticles

When the peptide FluPep ligand was included in the ligand mix to functionalise the nanoparticles, its molar fraction in percent in relation to the matrix ligand should reflect its grafting density on the gold nanoparticles [17,22,26,30–32]. This can be determined by chromatography targeting specifically the grafted function, which also provides a means to purify the functionalised gold nanoparticles from those not functionalised, when the molar fraction of the functional ligand is low. Thus, when 10% of the functionalised gold nanoparticles bind to the chromatography column, most of these (95%) will possess just one grafted functional ligand [26,30]. Since FluPep ligand, when incorporated into a nanoparticle ligand shell, has a net charge at pH 7.4 of +6, cation-exchange chromatography was used to purify the functionalised gold nanoparticles. Parallel chromatography was performed on the anion exchanger DEAE-Sepharose to control for possible non-specific binding of FluPep ligand to Sepharose.

**Figure 2:**
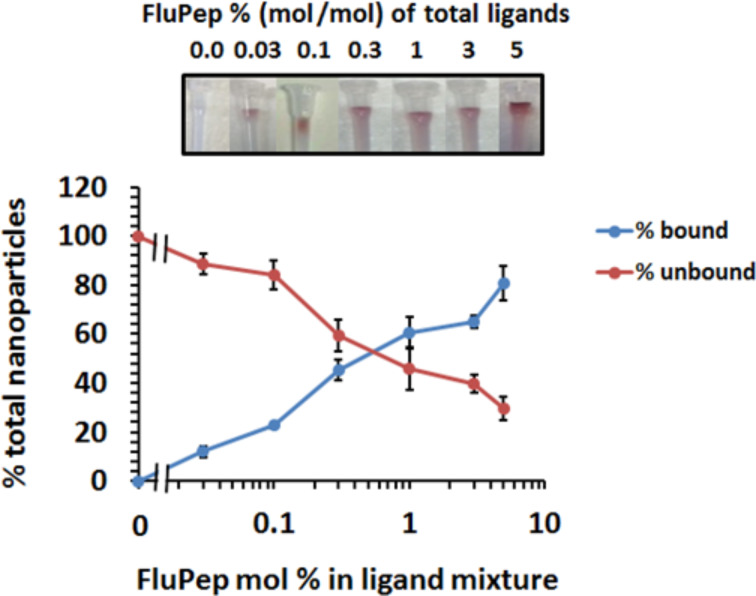
Purification of FluPep-ligand-functionalised gold nanoparticles by CM-Sepharose cation-exchange chromatography. Chromatography on CM-Sepharose was carried out with gold nanoparticles functionalised with different molar fractions of FluPep ligand. Top: images of columns after loading and washing with PBS. Bottom: quantification by absorption at 450 nm [18] of unbound (flow-through and PBS wash fractions) and bound (eluted with 2 M NaCl) fractions as a percentage of total nanoparticles applied to the column. Results are the mean ± SD (*n* = 3).

Mixed-matrix gold nanoparticles did not to bind to either CM-Sepharose or DEAE-Sepharose (Supporting Information File 1, Figure S2), as described previously [26]. Similarly, when FluPep ligand was incorporated in the ligand shell there was no binding to DEAE-Sepharose, indicating an absence of non-specific interactions with the chromatography resin (Supporting Information File 1, Figure S2). In contrast, the FluPep-functionalised gold nanoparticles bound to CM-Sepharose and were eluted by increasing electrolyte concentrations (Figure 2). Thus, the FluPep-functionalised gold nanoparticles ion-exchanged on this chromatography support, which is, therefore, suitable for their purification. Gold nanoparticles were synthesised with a range of molar fractions of FluPep ligand. After application of the gold nanoparticles to the column, the non-functionalised gold nanoparticles were collected in the flow-through and the functionalised ones were then eluted. Quantification of the gold nanoparticles by UV–vis spectrophotometry then allowed the relation of bound and unbound gold nanoparticles to the molar fraction of FluPep in the original ligand mixture to be analysed. The data indicate that at 0.03 mol %, 10% of the gold nanoparticles bound the column and thus most (ca. 95%) of these gold nanoparticles will possess just one single FluPep ligand [30]. At higher molar fractions the number of FluPep ligands per nanoparticle will increase. It is interesting to note that not all gold nanoparticles were observed to bind to the CM-Sepharose column at higher molar fractions of FluPep ligand, something that has been observed previously with other functional peptides [31–32].

### Anti-influenza activity of FluPep and FluPep ligand

**Figure 3:**
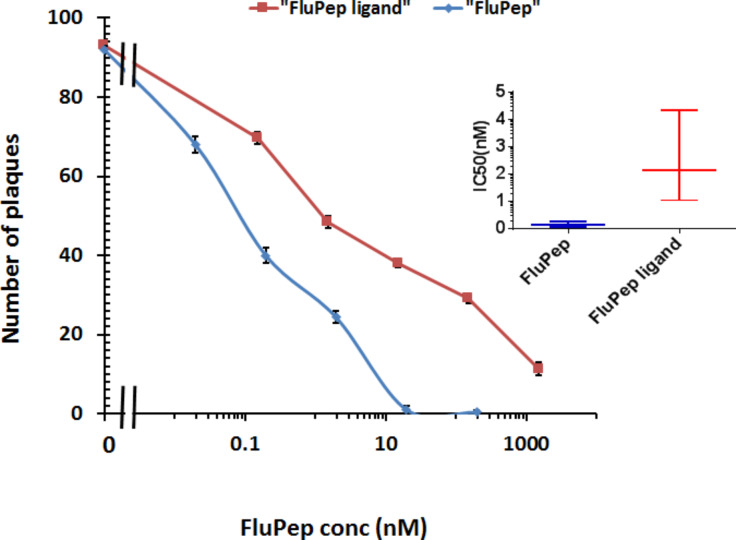
Determination of the half maximal inhibitory concentration (IC_50_) of FluPep and and FluPep ligand in a plaque assay. Inhibition of plaque formation as a function of the concentration of FluPep and FluPep ligand. Inset: IC_50_ values. Results are the mean ± SD (*n* = 3).

MDCK cells are susceptible to both influenza type-A and type-B viruses and are routinely used to measure influenza virus infectivity. The principle is that following influenza virus particle binding, the virion will enter the cell, replicate its genome, translate viral protein components, assemble viral particles and then egress from the infected cell by lysis. The released viral particles will then infect neighbouring cells. By putting an agarose overlay on cultured cells following incubation for 1 h with the virus to initiate infection, long-range diffusion of the virus is prevented, ensuring only neighbouring cells are infected upon cell lysis. Consequently, after three days an area of lysed cells is apparent, the so-called “plaque”, which is visualised as a clear circle that does not stain with toluidine blue. In control (no virus) and vehicle (DMSO)-treated cells, the cell monolayer in the well was evenly stained (Supporting Information File 1, Figure S3). In the presence of virus, there were substantial areas where cells had lysed (plaques) and there was no staining. Virus titre was adjusted so that the cleared lysed cell areas corresponded to individual plaques, i.e., clear circles that could be distinguished from one another and so be counted (corresponding to ca. 100 plaques/well). In the presence of FluPep there was a reduction in the number of plaques and this was concentration-dependent (Figure 3). Counting plaques in multiple experiments allowed for the determination of the dose response and the IC_50_ values. In these experiments the IC_50_ value of the original FluPep sequence was found to be of the order of 140 pM (Figure 3, inset). This is less potent than the value described in the original publication, where an IC_50_ value of 14 pM was measured [15]. The source of this discrepancy is unknown, but more than likely relates to differences in cells such as passage number, and/or virus preparations. The FluPep ligand, used to functionalise gold nanoparticles was slightly less potent, with an IC_50_ value of around 210 pM, suggesting that the N-terminal extension CVVVTAA reduced the antiviral activity to some extent (Figure 3).

### Anti-flu activity of FluPep-functionalised gold nanoparticles

**Figure 4:**
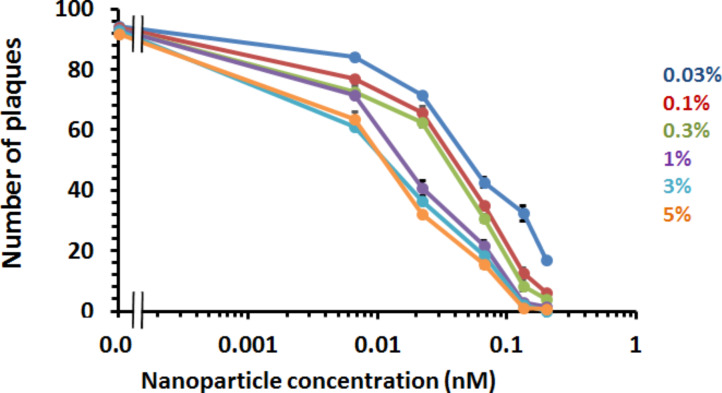
Effect of gold nanoparticles functionalised with FluPep ligand on influenza virus plaque formation. Inhibition of plaque formation as a function of the concentration of gold nanoparticles functionalised with different molar fractions of FluPep ligand in the ligand mixture. Results and the mean ± SD (*n* = 3).

The addition of mixed-matrix-passivated nanoparticles as control had no detrimental effect on viral infectivity, since their addition to the cells did not change the number of plaques. However, when purified mixed-matrix-capped gold nanoparticles functionalised with 5% (mol/mol) FluPep ligand were added, there was a marked decrease in the number of plaques, i.e., a reduced virus infectivity (Figure 4). Thus, antiviral activity of FluPep ligand was maintained when it was conjugated to gold nanoparticles.

For gold nanoparticles functionalised with 0.03% (mol/mol) FluPep ligand, the number of plaques started to decrease at 20 pM gold nanoparticles and reached a minimum of approximately two to eight plaques at 200 pM (Figure 4). As the grafting density of FluPep ligand was increased, so did the antiviral activity, to reach a maximum at 5% (mol/mol) FluPep (Figure 4). This is reflected by the decreased IC_50_ value, which shows a 4.5-fold greater potency of nanoparticles functionalised with 5% (mol/mol) FluPep ligand, when compared to nanoparticles functionalised with just 0.03% (mol/mol) FluPep ligand (Table 1). The nanoparticles were always purified by cation-exchange chromatography (Figure 2), so in all cases nanoparticles had at least one FluPep ligand. Taken together, these data indicate that the potency of FluPep ligand on the nanoparticles is greater than that of free FluPep ligand and of the native FluPep peptide (inset of Figure 3 and Table 1).

**Table 1:** IC_50_ values of gold nanoparticles functionalised with different molar fractions of FluPep ligand.

mol % FluPep ligand	IC_50_ (nM)

0.03%	0.073
0.1%	0.068
0.3%	0.058
1%	0.023
3%	0.016
5%	0.015

### Stability of FluPep functionalised silver nanoparticles

**Figure 5:**
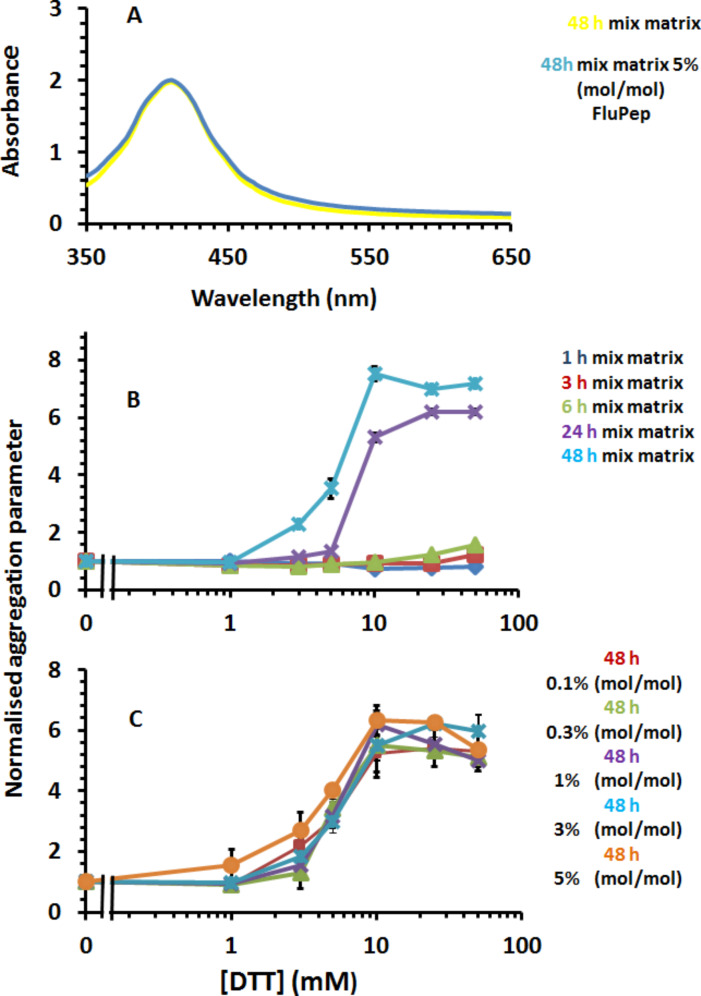
Stability of silver nanoparticles to DTT ligand exchange. (A) UV–vis spectra of mixed-matrix-capped silver nanoparticles and mixed-matrix-capped silver nanoparticles incorporating 5% (mol/mol) FluPep ligand in PBS. Time and dose-dependence of DTT ligand exchange for (B) mixed-matrix silver nanoparticles and (C) silver nanoparticles incorporating different molar fractions of FluPep ligand. Results are the mean ± SD (*n* = 3).

Silver has well-established antimicrobial properties [33]. It was of interest to determine whether FluPep-functionalised silver nanoparticles exhibited a similar anti-flu activity to their gold counterparts. The mixed-matrix ligand shell has been shown to impart good stability to silver nanoparticles [17]. First, the effect of incorporating FluPep into the ligand shell on the nanoparticle stability was measured. As for gold nanoparticles, up to 5% (mol/mol) FluPep ligand incorporated into the ligand matrix had no discernible effect on the handling and purification of the silver nanoparticles. The silver nanoparticles did not bind non-specifically to Sephadex G25, as they eluted in the void volume and their UV–vis spectrum in PBS was indistinguishable from that of control mixed-matrix silver nanoparticles (Figure 5A). When challenged by a small thiol, DTT, the silver nanoparticles passivated by the mixed-matrix ligand shell were somewhat more prone to ligand exchange than their gold counterparts. Thus, after 3 h and 6 h in 50 mM DTT a small increase in aggregation parameter was apparent (Figure 5B). After 24 h and 48 h, the aggregation parameter started to rise at 3 mM DTT, which was most pronounced at 48 h. Whereas an increase in aggregation parameter indicates a loss of ligand shell integrity and nanoparticle aggregation, the absolute value of aggregation parameter greater than 1 is not informative, since nanoparticle can aggregate in different ways that affect the UV-vis spectra differently. Thus, the inclusion of 5% (mol/mol) FluPep ligand in their ligand shell did not change the stability of the silver nanoparticles with respect to DTT-mediated ligand exchange (Figure 5C and Supporting Information File 1, Figure S4).

**Figure 6:**
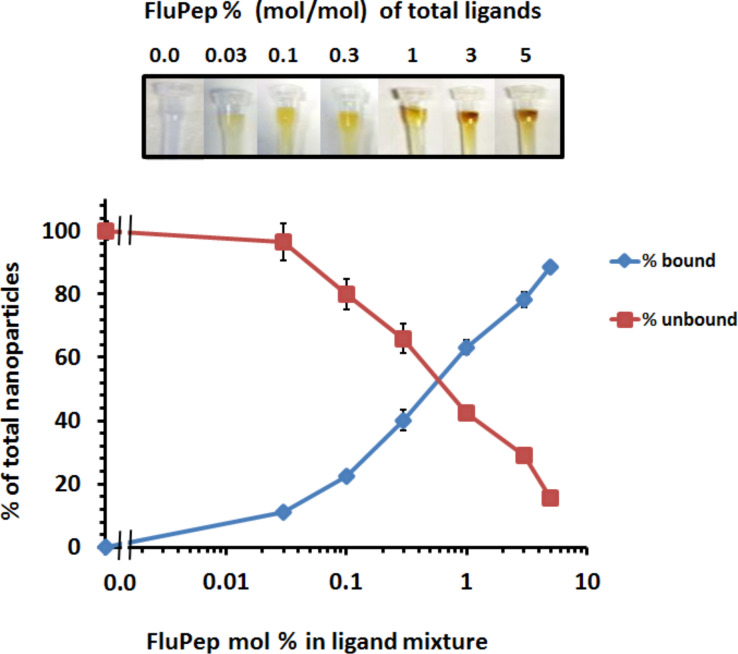
Purification of FluPep ligand-functionalised silver nanoparticles by CM-Sepharose cation-exchange chromatography. Silver nanoparticles functionalised with different molar fractions of FluPep ligand were subjected to chromatography on CM-Sepharose. Top: images of columns after loading and washing with PBS. Bottom: quantification of unbound (flow-through and PBS wash fractions) and bound (eluted with 2 M NaCl) fractions as a percentage of the total nanoparticles applied to the column. Results are the mean ± SD (*n* = 3).

Purification of the FluPep-functionalised silver nanoparticles was achieved by cation-exchange chromatography on CM-Sepharose. As the percentage of FluPep incorporated into the ligand shell increased, so did the percentage of nanoparticles bound to CM-Sepharose (Figure 6). This suggests that the molar fraction of FluPep ligand in the initial mixture of ligands added to the nanoparticles reflects its incorporation into the ligand shell [26,30]. Thus, as for gold nanoparticles, when 10% of the total nanoparticle preparation bound to the CM-Sepharose column, ca. 95% of the bound nanoparticles will possess a single FluPep ligand [30]; at higher molar fractions of FluPep ligand the silver nanoparticles will incorporate more than one FluPep ligand.

### Anti-influenza activity of FluPep ligand incorporated to silver nanoparticles

**Figure 7:**
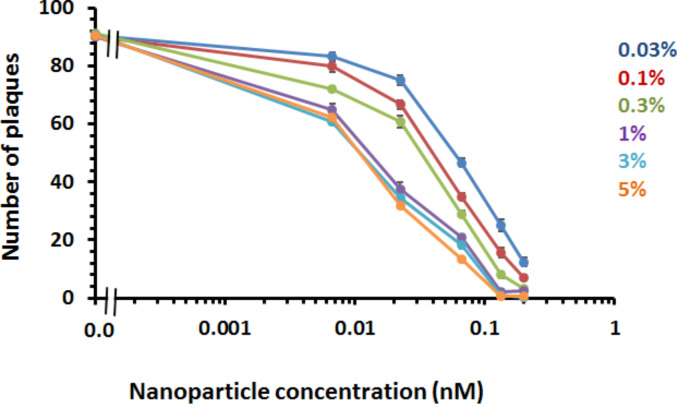
Determination of the half maximal inhibitory concentration (IC_50_) of silver nanoparticles functionalised with FluPep ligand. Inhibition of plaque formation as a function of the concentration of silver nanoparticles functionalised with different molar fractions of FluPep ligand. Results are the mean ± SD (*n* = 3).

Control mixed-matrix-passivated silver nanoparticles had no effect on viral infectivity (Figure 7). It is the silver (Ag^+^) ions that exert antimicrobial activity [33]. Thus, this result indicates that there is not a substantial release of Ag^+^ ions from the silver nanoparticles during the experiment. This concurs well with our data (Figure 5) and previously reported observations [17] demonstrating that the mixed-matrix ligand shell imparts good stability to the silver nanoparticles. In contrast, functionalisation of silver nanoparticles with FluPep ligand caused a marked reduction in number of plaques (Figure 7). As the molar fraction of FluPep ligand in the ligand shell increased, so did the antiviral activity of these particles (Figure 7). Around 10% of silver nanoparticles functionalised with 0.03% (mol/mol) FluPep ligand bind to the CM-Sepharose column, indicating that the majority of these will carry just a single FluPep ligand (Figure 6). These nanoparticles are as potent as free FluPep and more potent than free FluPep ligand (inset of Figure 3 and Figure 7B). Silver nanoparticles with a higher grafting density of FluPep ligand (so functionalised with a higher mol %) had a greater anti influenza virus activity than either of the free peptides (inset of Figure 3 and Figure 7B).

**Table 2:** IC_50_ values of silver nanoparticles functionalised with different molar fractions of FluPep ligand.

mol % FluPep ligand	IC_50_ (nM)

0.03%	0.14
0.1%	0.077
0.3%	0.056
1%	0.019
3%	0.016
5%	0.015

## Conclusion

We have demonstrated that FluPep, a peptide demonstrated to inhibit effectively influenza A virus subtypes, including H1N1, H3N2 and H5N1 [15] can be successfully incorporated into the ligand shell of gold and silver nanoparticles. The FluPep-functionalised nanoparticles have a greater antiviral activity than the free peptide. The FluPep amino acid sequence is hydrophobic and its solubilisation requires dimethyl sulfoxide (DMSO). Although a useful solvent, its use in therapies is problematic due to DMSO causing potential adverse reactions in some individuals such as a sensation of burning, vesiculation, dryness of skin and local allergic reactions [34–36]. PEGylation of peptides has been successfully used to increase their solubility and in some instances their biological half-life [37–38]. However, such modifications may alter the activity of the peptide and can have side effects due to cleavage products [39], and so whether this is a viable route for FluPep remains to be determined. Conjugation to nanoparticles is shown here to be another means to deliver effectively and safely FluPep ligand with enhanced activity in a solvent-free formulation.

In relation to the safety of a nanoparticle formulation of FluPep, the fate of nanoparticles delivered in murid rodents has been examined [40–47] and shows transfer of nanoparticles to vascular organs, including the brain. However, the concentrations of nanoparticles used in these experiments are ca. 1000-fold higher than used in the present work. Moreover, there is currently no conclusive in vivo evidence that the nanoparticles cross the blood–brain barrier. Incubation of peripheral blood mononuclear cells with citrate and mixed-matrix gold nanoparticles demonstrates that the mixed-matrix ligand shell markedly reduces the reaction of the peripheral blood mononuclear cells to the nanoparticles [48]. Therefore, whilst it remains to be determined experimentally, it would seem that the FluPep-functionalised nanoparticles may well be safe to deliver to target organs such the upper respiratory tract, a primary target for respiratory viruses including influenza. Although the mixed-matrix ligand shell largely prevents the dissolution of the silver, there are examples of ligand shells that allow solvent access to the silver and so the dissolution of the silver, at least under laboratory conditions [17]. Thus, it would be possible to design silver nanoparticles that act through FluPep ligand and released Ag^+^ ions. This would also reduce the potential for accumulation of nanoparticles in the body during repeated use of a FluPep-nanoparticle therapeutic. The pulmonary route to deliver drugs against respiratory infections is well established, so delivery of functionalised nanoparticles against flu viruses is highly feasible. Therefore, a nanoparticle formation of FluPep ligand or analogous peptides offers a route to new treatments for influenza and other respiratory pathogens.

## Experimental

### Materials

Peptides FluPep WLVFFVIFYFFRRRKK, FluPep ligand CVVVTAAAWLVFFVIFYFFRRRKK and the ligand shell matrix peptidol CVVVT-ol were purchased from Peptide Protein Research (PPR Ltd, Hampshire, UK). The alkanethiol ethylene glycol ligand, HS(CH_2_)_11_(OC_2_H_4_)_4_OH, was purchased from Prochimia (ProChimia Surface Sp. z o.o., Sopot, Poland). Gold nanoparticles of 9 nm diameter stabilized in citrate buffer were purchased from British Biocell (BBInternational Ltd, UK) and silver nanoparticles of ca. 10 nm diameter from nanoComposix Inc. (CA, USA). Nanosep filters with 10 kDa cut-off were from PALL (PALL Corp., Portsmouth, and Hants, UK). UV–vis spectra (2 nm incremental steps) were measured using a SpectraMax Plus spectrophotometer (Molcular Devices, Wokingham, UK) and 384-well plates from Corning (Lowell, US) and the concentration of gold nanoparticles and of silver nanoparticles was determined at 450 nm [18] and 392 nm [49], respectively.

### Synthesis of FluPep functionalised nanoparticles

Mixed-matrix ligands 70:30 (mol/mol) CVVVT-ol/ HS(CH_2_)_11_(OC_2_H_4_)_4_OH were prepared as described [26] by first diluting 35 µL CVVVT-ol (4 mM DMSO/H_2_O) with 35 µL ddH_2_O, and 6 µL HS(CH_2_)_11_(OC_2_H_4_)_4_OH (2 mM) with 6 µL EtOH and 18 µL H_2_O. Adding the two solutions together yielded a 2 mM ligand solution of 70% (mol/mol) CVVVT-ol and 30% (mol/mol) HS(CH_2_)_11_(OC_2_H_4_)_4_OH. To functionalise the nanoparticles with FluPep ligand, this was incorporated into the initial ligand mix at the molar fraction indicated in the figure legends. The ligand mixture was added to 900 µL (gold or silver) nanoparticles and vortex-mixed. Once mixed, 100 µL of 10× phosphate-buffered saline (PBS: 137 mM NaCl, 3 mM KCl, l.8 mM Na_2_HPO_4_, 15 mM KH_2_PO_4_) with Tween-20 (0.1% (v/v)) pH 7.4 was added to the gold nanoparticles [26] and 10× (100 mM NaNO_3_, 20 mM HEPES with Tween-20 (0.1% (v/v)) pH 7.4 to the silver nanoparticles [17], vortex-mixed and the (gold or silver) nanoparticles were placed on a rotating wheel for 24 h. Nanoparticles were concentrated 10-fold using 10 kDa Nanosep centrifugal filters (PALL Corp., Portsmouth, Hants, UK). Samples were then centrifuged for 7 min at 10000 rpm (ca. 12,000*g*) and the gold nanoparticles diluted with 1× PBST (PBS 0.05% (v/v) Tween-20) and silver nanoparticles with 1× (100 mM NaNO_3_, 20 mM HEPES). In the case of FluPep-functionalised nanoparticles, the separation of free FluPep ligand (*M*_w_ = 2967 Da) required six washes, each wash involving a 10-fold dilution of the nanoparticles on a 10 kDa cut-off Nanosep filter and centrifugation. The nanoparticles were then further separated from excess ligands by applying them (100 µL) to a 5 mL Sephadex G25 gel filtration column with PBS as a mobile phase.

### Ion-exchange chromatography

Ion-exchange chromatography was performed on custom-made mini columns of diethylaminoethyl (DEAE) and carboxymethyl (CM) Sepharose (GE Healthcare Bio-Sciences AB, Sweden). The gel slurry was packed into a white pipette tip (200 µL) using half the filter as a frit and equilibrated in PBS. Capped nanoparticles were concentrated and exchanged into the appropriate buffer using a 10 kDa cut-off Nanosep centrifugal filter. The nanoparticles were then applied to the column, the unbound fraction was recovered. Columns were washed with PBS and eluted with 1 M NaCl and then 2 M NaCl in 8 mM Na_2_HPO_4_, 15 mM KH_2_PO_4_, pH 7.4.

### Calculation of the aggregation parameter (AP)

The surface plasmon absorption peak of 8.8 nm diameter gold nanoparticles is at 520 nm. When gold nanoparticles are aggregated, their surface plasmons couple causing a red shift in their plasmon absorbance to approximately 650 nm. The aggregation parameter (AP) was defined as (*A*_650nm_ − *A*_ref 650nm_)/(*A*_520nm_−*A*_ref 520_), where *A*_650nm_ and *A*_520nm_ are the absorbance of gold nanoparticles at 650 nm and 520 nm, respectively, and *A*_ref 650nm_ and *A*_ref 520_ are the absorbance of water at 650 nm and 520 nm, respectively [27]. For comparison of results, this primary stability parameter was normalised by dividing the AP value of control ligand-capped gold nanoparticles measured in milli Q water where [DTT] = 0.

For silver nanoparticle diameters of approximately 10 nm, the surface plasmon absorption peak with a mixed-matrix ligand shell is at 410 nm. The AP for silver nanoparticle was defined as (*A*_600nm_ − *A*_ref 600nm_)/(*A*_410nm_ − *A*_ref 410_), where *A*_600nm_ and *A*_410nm_ are the absorbance of Ag nanoparticles at 600 nm and 410 nm, respectively and *A*_ref 600nm_ and *A*_ref 410_ are the absorbance of water at 600 nm and 410 nm, respectively.

It is important to note that values of aggregation parameters greater than 1 do not necessarily provide information on the degree of aggregation of the nanoparticles, since completely aggregated nanoparticles may exhibit different UV–vis spectra. For example, small aggregates of nanoparticles that remain in solution will show a red-shifted peak due to plasmon coupling, whereas larger aggregates that may settle may present a featureless UV–vis spectrum.

### Cell culture

Madin–Darby canine kidney epithelial cells [MDCK (ATCC CRL-2936)] were grown in Dulbecco’s modified Eagle’s medium (DMEM) supplemented with 5% (v/v) foetal calf serum (FCS) (Labtech International Ltd, East Sussex, UK), 1% (v/v) 200 mM L-glutamine, 1% (v/v) 100 U/mL penicillin and 1% (v/v) 100 µg/mL streptomycin (Gibco, Life Technologies, UK) and incubated in a humidified environment at 37 °C under 5% (v/v) CO_2_ atmosphere. Cells were detached with 0.05% (w/v) trypsin in the chelating agent, 1× Versene-EDTA (Gibco, Life Technologies, UK) and plated at a dilution of 1:4.

### Preparation of influenza virus stock

MDCK cells were grown to 90% confluence in T25 tissue culture flasks (VWR, Lutterworth, Leicestershire, UK), which corresponds to 7 × 10^6^ cells/flask. Then, the cell monolayer was washed with 2 × 5 mL PBS, and virus (A/WSN/33 H1N1 subtype) was added at a multiplicity of infection (MOI) of 0.001 in 2 mL DMEM. Cells were incubated with virus for 1 h at 37 °C on a rocking platform. Virus-containing medium was removed and the cell monolayer washed with 2 × 5 mL DMEM, then 5 mL *N*-acetyl trypsin (Sigma-Aldrich, Merck, UK), 2.5 µg/mL in DMEM, was added and incubated for 24–48 hours at 37 °C until a significant cytopathic effect had developed, to a point where the cells were lifting from the flask substrate. Medium was removed and centrifuged for 5 min at 2500 rpm to remove cell debris and the supernatant, which represented the viral stock, was stored at −80 °C.

### Virus plaque assay

MDCK cells were grown in 6-well plates (STARLAB international, Hamburg, Germany), 10^6^ cells/well) for two days. At confluence, monolayers of MDCK cells were then infected with a serial dilution of influenza virus inoculum (sufficient to obtain approximately 100 plaques per well) for 1 h at 37 °C on a rocking platform. An agarose overlay was prepared by mixing equal volumes of 2% (w/v) of pre-warmed (55 °C) low-melting agarose (Melford Laboratories Ltd, Blideston Road, Ipswich, UK) and the overlay solution (14 mL 10× MEM, 3.7 mL 7.5% (w/v) bovine serum albumin (fraction V, Sigma-Aldrich, UK), 1.4 mL L-glutamine, 2.6 mL 7.5% (w/v) NaHCO_2_, 1.4 mL 1 M HEPES, 1.4 mL (1% (v/v) 100 U/mL penicillin and 1% (v/v) 100 µg/mL streptomycin), 44.8 mL H_2_O and 5 µL *N*-acetyl trypsin) to give a final 1% (w/v) agarose mixture. After 1 h of incubation of the cells with the virus, the supernatant was removed from the plates and overlaid with 2–3 mL of the 1% (w/v) agarose overlay solution. The plates were left at room temperature for 15 min for the overlay to solidify and then inverted and placed in an incubator at 37 °C, 5% (v/v) CO_2_ for 3 days for plaques to develop. Cells were then fixed with 4 mL 10% (v/v) neutral buffered formalin (Leica Biosystems Peterborough Ltd, Bretton Peterborough, Cambridgeshire) for 1 h, after which the formalin and overlay were removed and cells were stained with 0.1% (w/v in water) toluidine blue, rinsed in water, and left to dry before counting plaques.

Virus titre was determined for each preparation of virus. The virus titre, as plaque-forming units (PFU) per mL, was determined by serially diluting the virus stock and counting the number of plaques in duplicate wells of MDCK cells. Only wells containing between 10 and 100 plaques were counted to ensure the assumption that each plaque formed was due to one infective virus particle was met.

## Supporting Information

File 1 Additional experimental data.
