# Supplementary material for: Enhanced inhibition of influenza virus infection by peptide–noble-metal nanoparticle conjugates
Source: Beilstein J Nanotechnol. 2019 May 14;10:1038–47. doi: 10.3762/bjnano.10.104 (PMC6541353; doi:10.3762/bjnano.10.104)
Supplement: File 1 — Additional experimental data. [file Beilstein_J_Nanotechnol-10-1038-s001.pdf]

BEILSTEIN JOURNAL OF NANOTECHNOLOGY

Supporting Information

for

Enhanced inhibition of influenza virus infection by peptide–noble-metal nanoparticle conjugates

Zaid K. Alghrair, David G. Fernig and Bahram Ebrahimi

Beilstein J. Nanotechnol. **2019,** *10,* 1038–1047. doi:10.3762/bjnano.10.104

Additional experimental data

License and Terms: This is a supporting information file under the terms of the Creative Commons Attribution License (http://creativecommons.org/licenses/by/4.0). Please note that the reuse, redistribution and reproduction in particular requires that the authors and source are credited.
The license is subject to the *Beilstein Journal of Nanotechnology* terms and conditions: (https://www.beilstein-journals.org/bjnano)

Table of contents

Figure S1:	Stability of gold nanoparticles functionalised with FluPep ligand	S3
Figure S2:	CM-Sepharose and DEAE-Sepharose chromatography of nanoparticles	S5
Figure S3:	Plaque assay example	S6
Figure S4:	Stability of silver nanoparticles functionalised with FluPep ligand	S7

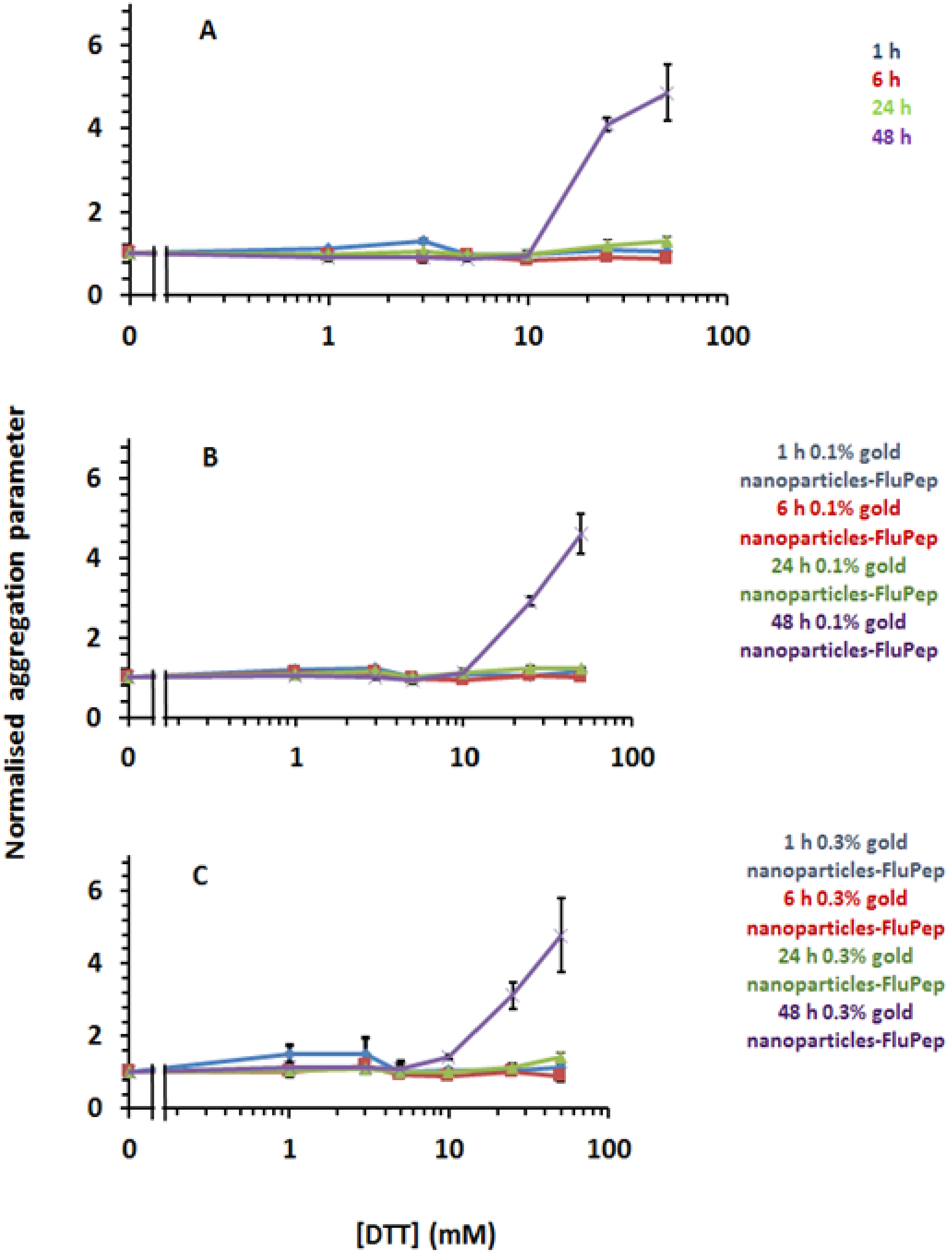
A
B
C
1 h
6 h
24 h
48 h
1 h 0.1% gold nanoparticles-FluPep
6 h 0.1% gold nanoparticles-FluPep
24 h 0.1% gold nanoparticles-FluPep
48 h 0.1% gold nanoparticles-FluPep
1 h 0.3% gold nanoparticles-FluPep
6 h 0.3% gold nanoparticles-FluPep
24 h 0.3% gold nanoparticles-FluPep
48 h 0.3% gold nanoparticles-FluPep
Normalised aggregation parameter
[DTT] (mM)
0
2
4
6
0
1
10
100

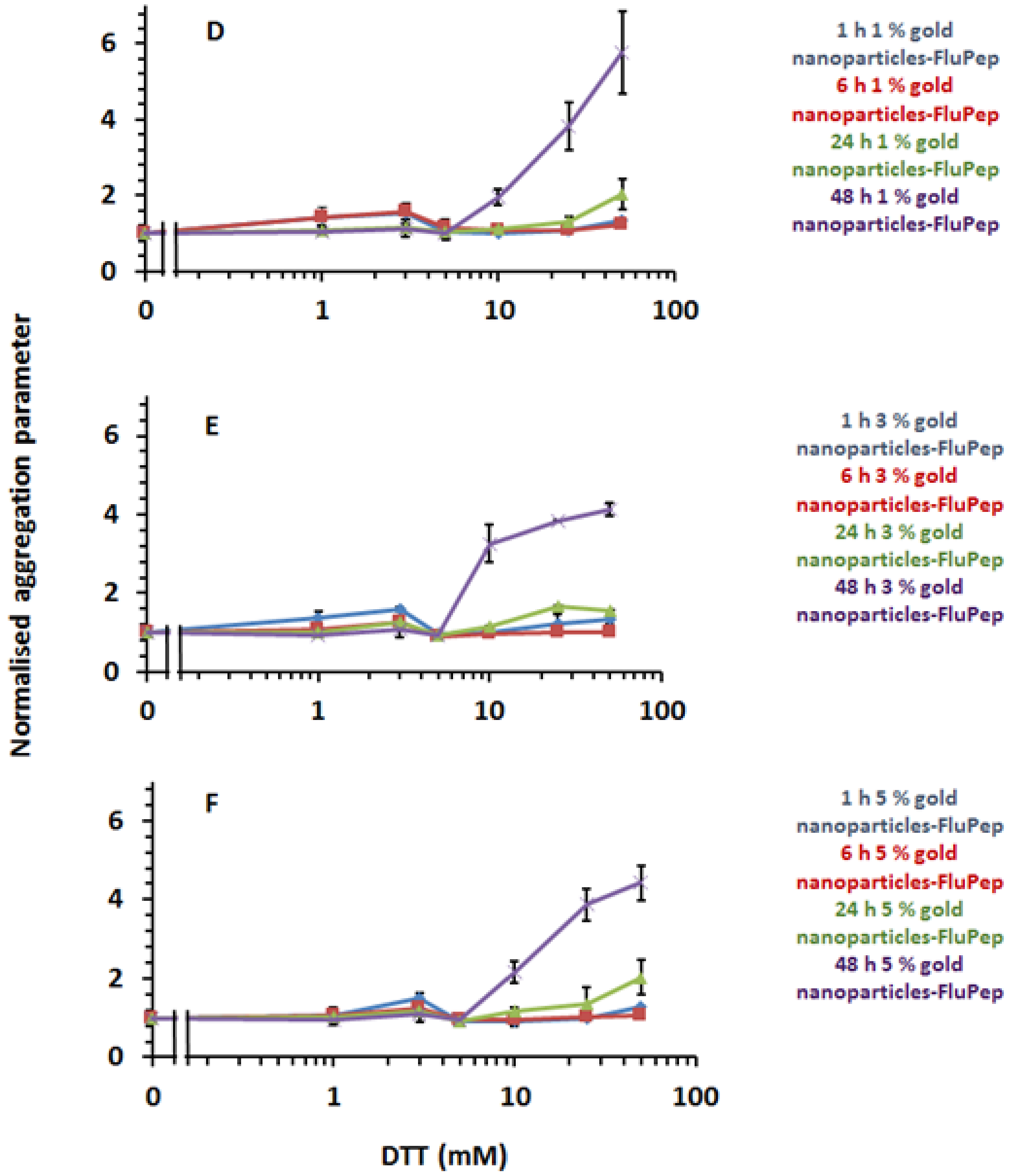

Figure S1: Stability of gold nanoparticles functionalised with FluPep ligand. Gold nanoparticles incorporating different molar fractions of FluPep ligand in their ligand shell were incubated in varying concentrations of DTT for different times and the normalised aggregation parameter was calculated. (A) 70:30 CVVVT-ol/$HS(CH_2)_{11}(OC_2H_4)_4OH$ mixed matrix gold nanoparticles. (B) 0.1% (mol/mol) FluPep ligand-functionalised gold nanoparticles. (C) 0.3% (mol/mol) FluPep ligand-functionalised Gold nanoparticles. (D) 1% (mol/mol) FluPep ligand-functionalised gold nanoparticles. (E) 3% (mol/mol) FluPep ligand-functionalised gold nanoparticles. (F) 5% (mol/mol) FluPep ligand-functionalised gold nanoparticles. Results are the mean ± SD (n = 3).

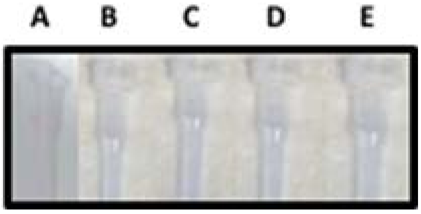

Figure S2: CM-Sepharose and DEAE-Sepharose chromatography of gold nanoparticles. Gold nanoparticles were applied to mini columns of CM- and DEAE-Sepharose, as described in "Experimental" in 20 mM Tris·HCl (pH 7.2) and the column was washed with PBS. (A) Mixed-matrix gold nanoparticles applied to DEAE Sepharose. (B) Mixed-matrix gold nanoparticles applied to CM Sepharose. (C) Mixed-matrix gold nanoparticles functionalised with 0.3% (mol/mol) FluPep ligand applied to DEAE Sepharose (D) Mixed-matrix gold nanoparticles functionalised with 3% (mol/mol) FluPep ligand applied to DEAE Sepharose. (E) Mixed-matrix gold nanoparticles functionalised with 5% (mol/mol) FluPep ligand applied to DEAE Sepharose.

When nanoparticles bind to ion-exchange columns, the concentrate in the top of the stationary phase, giving a clear red (gold, Figure 2) or yellow (silver, Figure 6) colour. In Figure S2A–E there is no evidence for gold nanoparticles bound to the top of the stationary phase, since this remains white. This demonstrates that the mixed-matrix passivated nanoparticles do not bind to CM- or DEAE-Sepharose and that when these nanoparticles are functionalised with FluPep ligand, they fail to bind to DEAE-Sepharose.

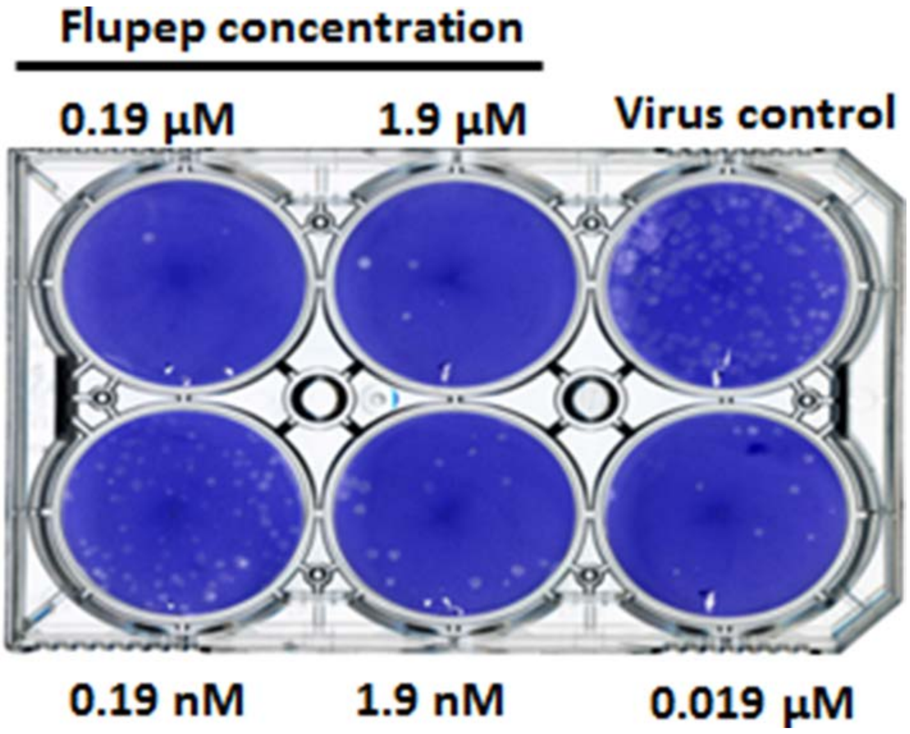

Figure S3: Example of a plaque assay. A confluent monolayer of MDCK cells was infected with influenza virus at varying dilutions and covered with an agarose overlay, to prevent the virus infection from spreading indiscriminately, as described in “Experimental”. Virus plaques are the cleared circles. A viral plaque is formed when a virus infects a cell within the fixed cell monolayer. The virus-infected cell will lyse and spread the infection to adjacent cells where the infection-to-lysis cycle is repeated. Here, the ability of FluPep to inhibit replication of WSN virus (ca. 100 plaques per well in control) was determined. Assays were carried out with virus in the presence of vehicle (DMSO, 1.5% final concentration), or virus in the presence of increasing concentrations of FluPep. Concentrations of FluPep are shown.

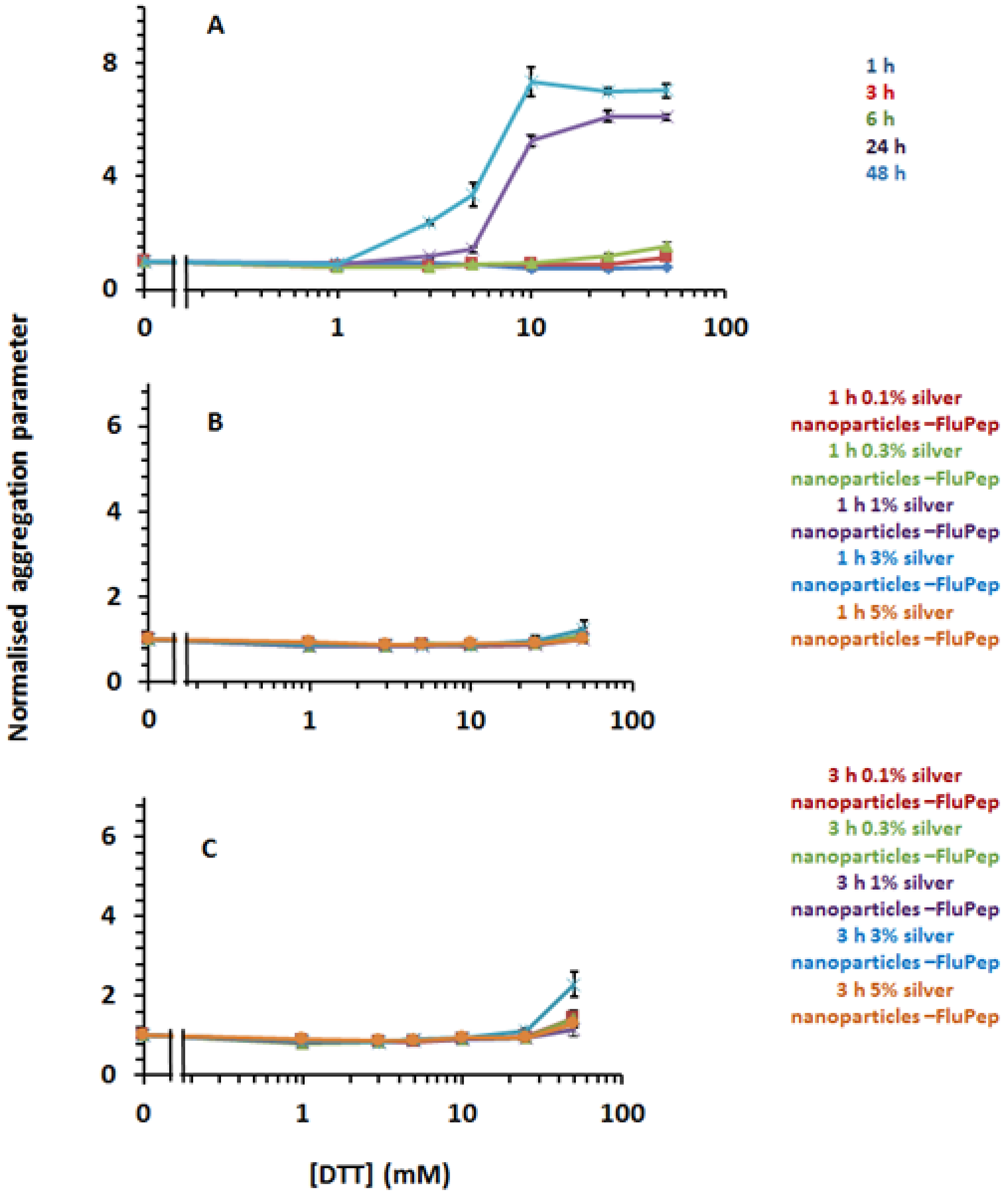
A
B
C
Normalised aggregation parameter
[DTT] (mM)
0
1
10
100
1 h
3 h
6 h
24 h
48 h
1 h 0.1% silver nanoparticles−FluPep
1 h 0.3% silver nanoparticles−FluPep
1 h 1% silver nanoparticles−FluPep
1 h 3% silver nanoparticles−FluPep
1 h 5% silver nanoparticles−FluPep
3 h 0.1% silver nanoparticles−FluPep
3 h 0.3% silver nanoparticles−FluPep
3 h 1% silver nanoparticles−FluPep
3 h 3% silver nanoparticles−FluPep
3 h 5% silver nanoparticles−FluPep

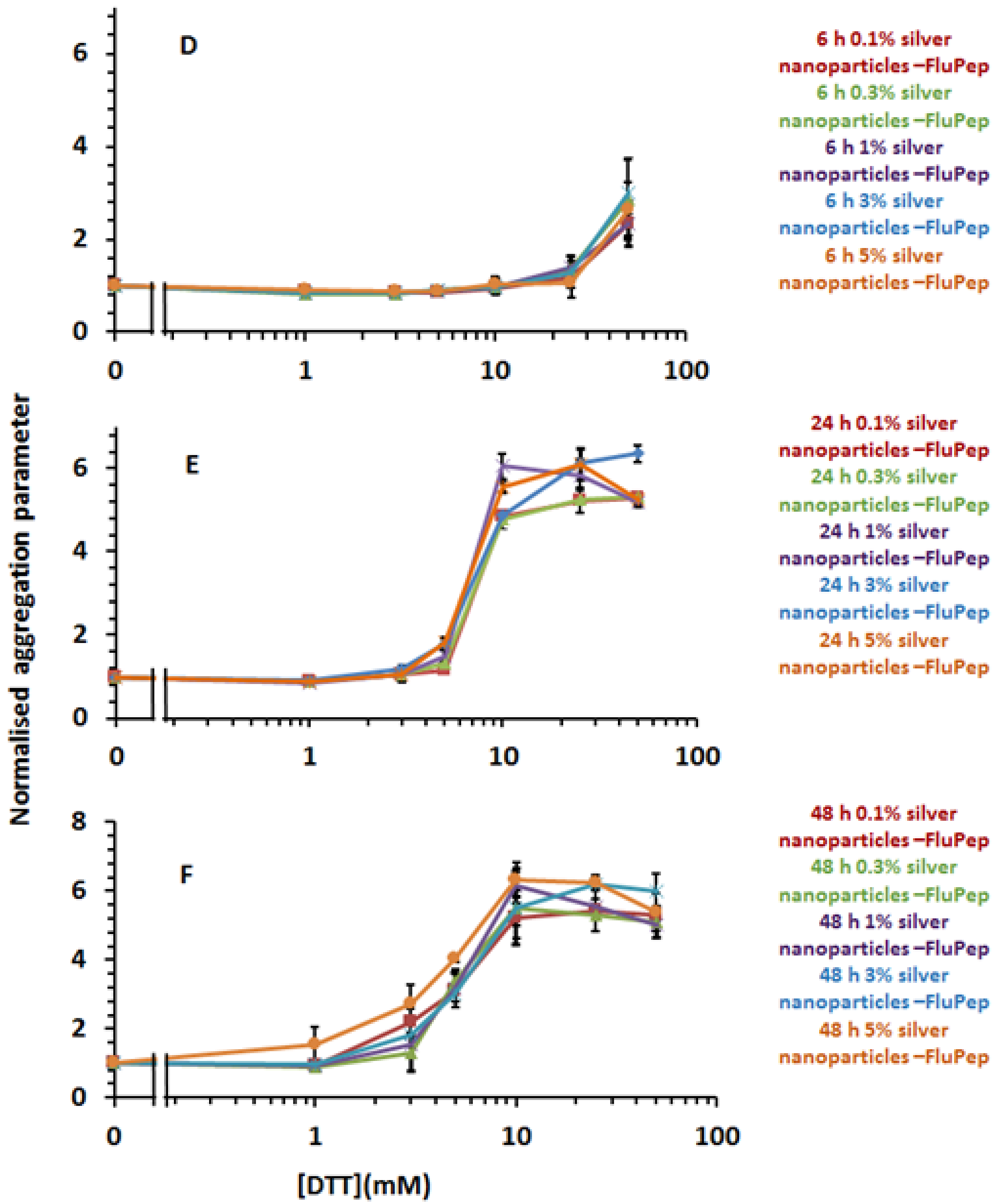

Figure S4: Stability of silver nanoparticles functionalised with FluPep ligand. Silver nanoparticles incorporating different molar fractions of FluPep ligand in their ligand shell were incubated in varying concentrations of DTT for different times and the normalised aggregation parameter was calculated. (A) 70:30 CVVVT-ol/$HS(CH_2)_{11}(OC_2H_4)_4OH$ mixed matrix silver nanoparticles. (B) 0.1% (mol/mol) FluPep ligand-functionalised silver nanoparticles. (C) 0.3% (mol/mol) FluPep ligand-functionalised silver nanoparticles. (D) 1% (mol/mol) FluPep ligand-functionalised silver nanoparticles. (E) 3% (mol/mol) FluPep ligand-functionalised silver nanoparticles. (F) 5% (mol/mol) FluPep ligand-functionalised silver nanoparticles. Results are the mean ± SD (n = 3).
